# PLGA Based "Lipid-Polymer Hybrid Nanoparticles": A Convergent Platform of Active and Targeted Drug Delivery

**DOI:** 10.34172/apb.46136

**Published:** 2026-05-23

**Authors:** Rajlaxmi Patro, Ch. Niranjan Patra, Subrata Mallick

**Affiliations:** ^1^School of Pharmaceutical Sciences, Siksha ‘O’ Anusandhan (Deemed to be University), Bhubaneswar 751003, India; ^2^Roland Institute of Pharmaceutical Sciences, Berhampur, India

**Keywords:** Lactic-glycolic acid polymer, “Nano Lipid-Polymer Hybrid”, Targeted drug delivery

## Abstract

A promising class of nano-carriers known as "Lipid-Polymer Hybrid Nanoparticles" (LPHNPs) combines the biocompatibility and functionality of lipid-based systems with the structural advantages of polymeric nanoparticles. This review summarizes structural classifications and preparation methods of PLGA-based lipid–polymer hybrid nanoparticles (LPHNPs). An exhaustive elaboration of various preparation techniques is provided, with an emphasis on their scalability and influence on physicochemical properties. Methods include solvent evaporation, nanoprecipitation, emulsion solvent evaporation, and homogenization, among others. To define the size of particles, morphology, surface charge, and thermal behavior, important characterization techniques such as differential-scanning-calorimetry, transmission electron microscopy (TEM), zeta potential (ZP), and particle size (PS) are described. Recent developments and therapeutic results are discussed here, followed by an extensive range of biomedical applications, spanning from drug delivery, gene therapy, and vaccine development to diagnostic imaging. In conclusion, the review highlights current challenges related to stability, repeatability, and targeted distribution. It also elaborates on potential future developments of personalized medicine, stimuli-responsive systems, and regulatory considerations. LPHNPs have the potential to serve as flexible platforms for the next generation of therapeutic and diagnostic modalities, potentially revolutionizing the field of nanomedicine.

## Introduction

 Nanoparticles (NPs) enhance drug delivery by improving solubility, enabling targeted release, and efficiently crossing biological barriers due to their small size and high surface area.^1,2^ Nanomedicine has advanced significantly over the past 20 to 30 years, and there is a growing effort to make it commercially available worldwide.^3,4^ NPs serve as nucleic acid (DNA or RNA) carriers in gene therapy applications. The advancement of gene therapy is intricately linked to the innovative application of nanoparticle technology.^5^ However, their current form is ineffective due to issues with side effects, degradation, and inadequate absorption.^6^ Among the various approaches, lipid-based nano-formulations are gaining popularity because of their unique ability to deliver both hydrophilic and lipophilic drugs. Lipid-based nanoformulations can enhance drug stability from physical, chemical, and enzymatic degradation; modulate drug release for controlled drug release and targeting; provide high drug loading; and improve absorption and bioavailability, biocompatibility, and reduced toxicity.^7,8^ Creating a carrier that can be encased, shielded, and released under particular, desirable circumstances is one technique.^9^ Lipid-based carriers have been developed for this purpose, including emulsions, liposomes,^10^ solid-lipid nanoparticles-(SLNs),^9,11^ as well as nanostructured-lipid-carriers(NLCs).^12,13^ Yumna Zaheer et.al in 2025 developed a solid lipid nanoparticle of Naringenin whose low bioavailability and poor absorption limit its therapeutic use. By using natural fatty acids, stearic acid, and lauric acid as a lipid and following the hot melt encapsulation preparation method, they found a 9-12 fold higher oral bioavailability of Naringenin.^9^ Lorena et.al 2025 did research on cancer therapy and reduced the adverse reaction of the treatment by NLC co-encapsulating Apigenin and Melatonin, which have low aqueous solubility and photosensitivity. To boost the bioavailability and effectiveness, they employed rosehip oil. Consequently, they were able to establish an additive pharmacological action against NLC that was individually loaded.^14^ Lipid-based drug carriers are challenging to administer because lipase’s enzymatic hydrolysis quickly eliminates particles, which resulting in the loss of some bioactive compounds, which results in instability of the structure.^15,16^

 Another emerging nano-based drug delivery system is polymeric nanoparticles, which have the potential of controlled release, biocompatibility, biodegradability, surface versatility, and targeting capabilities.^17,18^ Gulsel et.al 2022 formulated oseltamivir phosphate-based polymeric nanoparticles based on poly(lactic-co-glycolic acid) (PLGA) for lung cancer treatment, where pegylated PLGA, a biodegradable polymer, is being used to target the cancer cells.^19^ By demonstrating a steady and/or regulated release of active molecules that fight cancer, polymer-based nanoparticles have shown great potential to overcome the challenges associated with cancer treatment. The advantages of using NPs as anticancer drug carriers include increased cellular uptake, enhanced encapsulation ability, improved pharmacokinetics, and bio-distribution, and superior specificity for malignant cells due to surface decorations and charge.^20-22^ However, the drug loading of polymeric nanoparticles is still challenging.

 As demonstrated by Zaheer et al. (2025) and Lorena et al. (2025), lipid-based carriers like SLNs and NLCs enhance drug bioavailability and therapeutic efficacy. However, NLCs provide greater stability and higher drug loading but lack standardized preparation techniques and thorough in vivo evaluation, whereas SLNs are more likely to experience drug expulsion and burst release. The controlled release and targeted delivery offered by polymeric nanoparticles, such as PLGA-based systems (Gulsel et al, 2022), are hindered by issues like complicated surface modifications and restricted drug loading. With few direct comparisons between lipid and polymer-based systems, these studies highlight important gaps in stability, drug loading, and in vivo performance, leaving open questions about their relative clinical advantages.

 Although lipid-based nanocarriers offer many advantages, such as improved therapeutic drug trapping efficiency and cost-effective manufacture, they also frequently show significant polydispersity, fast load release, and decreased stability. Additionally, the restricted chemical change capacity of these nanostructures restricts their application in active targeting techniques. Instead, polymeric nanosystems have a high potential for chemical alterations but are challenging during the drug-loading step.^23,24^ To overcome these limitations, LPHNPs integrate biodegradable polymers with natural lipids, combining the benefits of both systems.^25^ While avoiding the drawbacks of each distinct system, the design of matrices integrating lipid and polymer-based systems may offer distinctive advantages. LPHNPs, a novel class of nanosystems, have therefore been discovered as a result of the idea of hybridizing two nanocarriers, i.e., a polymer structure (core) encased in a lipidic shell. These nanoparticles were created to provide the distinctive qualities of systems based on lipids and polymers.^26^ The “HYBRID” term was used as the NPs combine the advantageous qualities of both polymeric and lipid nanoparticles ^27^. The lipid enhances drug loading and membrane permeability, while the polymer regulates the quantity of drug released. Their potential for usage is significantly augmented in situations of insufficient distribution by their capability to enrich both biocompatibility and physical stability. The LPHNPs mainly have three distinctive layers, the structure contain:

- A biodegradable, natural, nonpolar polymer core like PLGA
- An external lipid-polyethylene glycol “shell” conjugation that can hold hydrophilic medications and inhibit the reticuloendothelial system uptake
- Lipids make up the third layer, which serves as a barrier between the outer and inner shells on either side like stearic acid, lauric acid, GMS, and soya lecithin.

Many researchers have done research and formulated LPHNPs with different drug molecules, polymers, and lipids, and achieved a greater degree of drug targeting to the target site, with higher oral bioavailability which as shown in Table 1. A promising platform is provided by LPHNPs for diagnostic imaging, targeted drug administration, and therapies by combining the advantages of biocompatibility, stability, adaptability, imaging capabilities, manufacturability, and reduced immunogenicity. These qualities make them desirable contenders for a variety of biological uses.^28-30^

**Table 1:** Literature review on composition of LPHNP

**Drug**	**Therapeutic category**	**T ½** **(h)**	**BCS class**	**Objective**	**Lipid**	**Polymer**	**Surfactant and co- surfactant**	**Ref.**
Curcumin Demethoxycurcumin (DMC) Bisdemethoycurcumin (BDM)	Antioxidant	6- 7	BCS-IV	To improve their bioactivities and antioxidant property	Cocoa butter CetylPalmitate Stearic acid Cholesterol CetylPalmitate	Pluronic F-127	Tween 80 and Span 80	^31^
Paclitaxel	Anticancer	3- 53	BCS-IV	Enhance the drug release	Bees wax	Bovine serum albumin (BSA)/dextran	Serine protease	^32^
Docetaxel	Anticancer (Prostate cancers)	17	BCS-IV	Tumor targeting	Phospholipids Cholesterol DSPE-PEG2000	PLGA PEG	PVA	^33^
Fisetin Bioactive phytochemical	Severe acute pancreatitis (SAP)	0.09	Class II	Sustained release	Gelucire, Labrasol	Chitosan	Tween 80	^34^
Iloprost (Ilo)	Vasodialtors Pulmonary arterial hypertension (PAH)	3–5 min	BCS Class II	development of controlled – release formulations to avoid multiple dosing	1,2-dipalmitoyl-sn-glycero-3-phosphocholine (DPPC) 1,2-distearoyl-sn-glycero-3-phosphoethanolamine-N-[methoxy (polyethylene glycol)-2000] (ammonium salt) (DSPE-PEG2000)	Poly(D, L-lactide-co-glycolide) (50/50) acid terminated (PLGA)	DSPE-PEG2000	^35^
Atorvastatin calcium	Atherosclerosis	14hr	BCS Class II	Enhancement of Bioavailability	Phospholipon 90 G	PLGA and PVA	Poloxamer 188	^36^
Doxorubicin with all trance retinoic acid	Anticancer	1-3 hr	BCS Class III	Enhancement of drug release	Phospholipid	PLGA Carboxymethyl DX, Dextran	PEG- DSPE	^37^
Hydrocortisone acetate	Anti-inflammatory	1.5 to 2 hours	Class II	Increase in epidermal permeation with very minimal dermatotoxicity.	Phospholipid	Polycaprolactone	Tween 80	^38^
Ribociclib	Anticancer	32 hours	Class IV	Enhancement of oral bioavailability	Phospholipids	PLGA	Poloxamer 188	^39^
Salinomycin	Chemotherapeutic drug	2 to 8 hours	BCS Class IV	Target to epidermal growth factor receptor	DSPE-PEG-Mal DSPE-PEG CFPE (1,2-dioleoyl-sn-glycero-3-phosphoethanolamine-N-carboxyfluorescein)	Poly(d, l-lactide-co-glycolide) (PLGA)	Soybean Lecithin	^40^
Doxorubicin (DOX)	Anticancer	1-3 hr	BCS Class III	To enhance the oral bioavailability.	Oleic Acid Stearic Acid (SA)	Eudragit Ethyl Cellulose	Sodium Lauryl Sulfate (SLS)	^41^
Tripterine	anti-inflammatory	2 to 8 hours	Class IV	Sustained release and combined therapeutic effect with phytomedicines.	Soybean Lecithin (Phosphatidylcholine > 90%) 3,3'-Dioctadecyloxacarbocyanine Perchlorate (DiO)	Poly(lactic-co-glycolic acid) (PLGA 75:25) Dextran Sodium Sulfate (DSS)	Sodium Selenite (Na₂SeO₃) Chlorpromazine	^42^
Baicalin	Anticancer	1 to 2 hour	BCS Class III	To target the colon	Soya Lecithin 1,2-Distearoyl-sn-glycero-3-phosphoethanolamine-N-[amino(polyethylene glycol)-2000] (DSPE-PEG2000)	Poly(lactic-co-glycolic acid) (PLGA) Chitosan	Poloxamers Tween 80	^43^
Itraconazole (ICZ)	Azole antifungals	20 - 60	BCS Class II	Dissolution enhancement of ICZ	GlycerylMonostearate (GMS)	Chitosan (CS)	Poloxamer 188	^44^
Methotrexate	Chemotherapeutic agent	3 to 10	BCS Class III	Controlled release	Phosphatidylcholine	PLGA Trypsin-EDTA	2',7'-Dichlorofluorescein Diacetate (DCFDA) Propidium Iodide (PI)	^45^
Posaconazole	Antifungal	15-35 hr	BCS-II	Improved bioavailabilty from 47.5% to 84.5% (rat intestine) Invitro drug release – 18hr Stability- 45 days	Stearic acid Span 60	Poly decalactone	Poloxamer 188	^46^

 This review article’s goal is to make a thorough review of types, methods of preparation, characterization techniques, and applications of LPHNPs with case studies. It aims to highlight the synergistic benefits of combining lipids and polymers, such as enhanced biocompatibility, improved drug encapsulation, controlled release, and increased stability. The review further explores various fabrication techniques and their impact on the physicochemical properties and performance of LPHNPs in drug delivery applications. By consolidating current findings, this article seeks to identify key challenges, propose solutions, and guide future research directions for the development of more effective and targeted nanocarrier platforms.

## Various Types of LPHNPs

###  Monolithic LPHNPs

 Monolithic LPHNPs, which are simply coupled nano-systems of lipids and polymers or copolymers with the help of surfactants, are the most fundamental kind. In this system, lipids are distributed across a polymeric/copolymeric matrix.^23^ To assess their in vivo anti-sporiatic effectiveness, Sudeep et al. developed monolithic LPHNPs laden with vitamin D3 in 2021. They demonstrated spherically structured NPs with a smaller particle size of 123.1 nm, PDI ~0.23, and EE ~77% by using the heat homogenization approach to get the therapeutic benefit in an imiquimod induced psoriasis mouse model through improved PASI scores and histological recovery. There are still a number of translational gaps in spite of these encouraging findings. Dose-dependent toxicity, possible systemic absorption, and long term dermal safety all essential for moving closer to clinical application are not covered in this study. Furthermore, there is no information provided regarding the preparation technique’s reproducibility or scalability, nor stability profiling under prolonged storage conditions. Lastly, there is still uncertainty about the best lipid/polymer ratios and formulation parameters for long-term efficacy due to the lack of mechanistic insights into skin penetration and retention. Therefore, to enhance the clinical potential of monolithic LPHNPs, future research should concentrate on thorough safety assessment, pharmacokinetic evaluation, and scale-up feasibility.^47^ Monolithic LPHNPs are the lipid-based and polymer-based nanoparticles of combined benefits. Polyethylene glycol molecules are scattered over a polymeric core-matrix composed of drug molecules in these lipids or lipid-NPs.^48^

###  Lipid Shell Nanoparticles with a Polymer Core

 A polymeric core enclosed in a lipid matrix makes up polymer-core lipid-shell hybrid NPs. Hydrophobic medications are better encapsulated, and their systemic metabolism is inhibited by the polymeric core. The lipid coat aids in controlling drug release and improves biocompatibility. The lipid coat’s amphiphilic lipids help to stabilize the matrix.^28^ Elkateb et al. stated that hybrid poly(lactic co-glycolic acid) nanoparticles protected by soy-lecithin that contained darunavir in addition to ritonavir for the treatment of Human Immunodeficiency Virus. They showed that phospholipids, like lecithin, stabilized LPHNPs through electrostatic stabilization. Although this architecture effectively shields medications from systemic metabolism and improves delivery, there are still some important gaps, such as the lack of long-term safety and in-vivo pharmacokinetic data, the possible immunogenicity of lipid components, and difficulties with large-scale reproducible manufacturing. Future research should address the feasibility of scaling up for clinical use as well as converting these in vitro and ex vivo benefits into strong in vivo efficacy and safety profiles.^49^

###  L-P-L Layered NPs

 L-P-L layered NPs are composed of a distinctive nanoparticulate assembly matrix consisting of layer by layer of lipid, polymer, and lipid covering the interior empty layer of lipid in the core.^50^ Such nanoparticles’ numerous layers effectively protect the medication from the environment and encourage improved encapsulation efficiency. There may be one or more lipid layers encircling this polymeric core.^51^ They are often formed by combining the characteristics of polymeric nanoparticles with liposomes to create complexes of lipids and polymers where a lipid bilayer or lipid multilayer envelops the polymeric-core surface. Aqueous buffer or water is typically used to fill the space between the core of the polymer and the lipid layer. To encourage electrostatic interactions with oppositely charged polymers, cationic or zwitterionic phospholipids were mostly used to prepare the coating for the lipoparticles.^52^ Najamsahar et al. in 2025 reported that Solid lipid nanoparticles containing sorafenib were covered with a cationic, biodegradable polymer. Lipid-core-chitosan shell hybrid sorafenib NPs were formed as a result of the electrostatic interactions between the negatively charged SLNs and cationic chitosan. The sorafenib SLN negative zeta potential (-18.6 mV) is inverted to a positive zeta potential (+ 21.2 mV), verifying their effective use of chitosan for surface coating. The chitosan-coated sorafenib SLNs were shown to have dramatically increased systemic exposure to SRF after oral administration to rats. Although this multilayer approach has the potential to enhance oral absorption and drug stability, there are still certain drawbacks, such as the difficulty of manufacturing, possible variations in layer homogeneity, difficulties with scalability, and a lack of knowledge regarding the long-term safety or immunogenicity of cationic coatings. Standardizing layer assembly, assessing chronic toxicity, and improving for clinical translation while preserving increased bioavailability should be the main goals of future studies.^53^

###  Biomimetic Lipid Polymer Hybrid Systems 

 Nanoparticles disguised inside erythrocyte cell membranes to replicate the surface chemistry of erythrocytes make up biomimetic hybrid nanoparticulate systems. Erythrocyte membrane coating improves hemocompatibility and stops the reticuloendothelial system from recognizing and absorbing it. These result in longer circulation time, decreased systemic metabolism, and enhanced in vivo stability.^54^ Biomimetic Lipid Polymer Hybrid Nanoparticle systems use stem cells, platelets, WBCs, and other elements to achieve immunological evasion, site specificity, prolonged circulation, and biological barrier traversal.^55^ Sun’s research team has concentrated on the possibility of hybrid iron oxide nanoparticles made of platelet and cancer stem cell membranes for photo-thermal treatment of head and neck squamous cell carcinoma. Together with magnetic resonance imaging and photothermal therapy, the hybrid nanoparticles demonstrated the capability to evade the immune system, extend the time of circulation, target malignancy, and limit tumor growth.^56^ Complex manufacturing procedures, possible membrane coating variability, immunogenicity risk with non-autologous membranes, and a lack of long-term in vivo safety data are some of the obstacles that still exist despite these benefits. To fully realize the therapeutic potential of biomimetic hybrid nanoparticles, future studies should concentrate on standardizing membrane isolation and coating processes, evaluating chronic toxicity, and investigating scalability for clinical translation.^57^

###  Polymer Caged Liposomes

 Liposomal carriers have made significant strides in delivering medications, peptides, nucleic acids, and other substances to their intended location. They have successfully passed over several physiological obstacles. However, a few drawbacks of liposomes, such as decreased stability in biometrics and storage, vulnerability to physicochemical reactivity, etc., limit their ability to reach their full potential.^58^ Researchers have used stimuli-responsive polymers to control medication release, taking advantage of this type of polymer.^59^ Sang-min Lee and his colleague 2011 derived two pharmacologically active types of cargo (Aslll and Nill) co-encapsulated in the liposomal core that are released when cholesterol-terminated poly-(acrylic acid) (chol-PAA) is added to the liposome using the post-insertion method and crosslinked with 2,20-(methylenedioxy)-bis(ethylamine). This creates a pH-sensitive polymer cage. Because the free acrylate groups are protonated at low pH, the polymer cage undergoes a stimuli-responsive conformational change that disrupts the lipid membrane and results in the chemomechanical release feature. This approach maximizes therapeutic efficacy while reducing off-target effects by fusing the tunable release profiles of polymers with the biocompatibility and targeting potential of liposomes. The intricacy of creating polymer-liposomes, the possible immunogenicity of polymer coatings, and scalability for clinical translation are still obstacles, though. For polymer-cages liposomal systems to fully realize their translational potential, future research should concentrate on improving polymer chemistry, analyzing long-term in vivo safety, and evaluating performance under physiological conditions.^60^ Figure 1 represent the schematic diagram of different types of LPHNPs.

**Figure 1:**
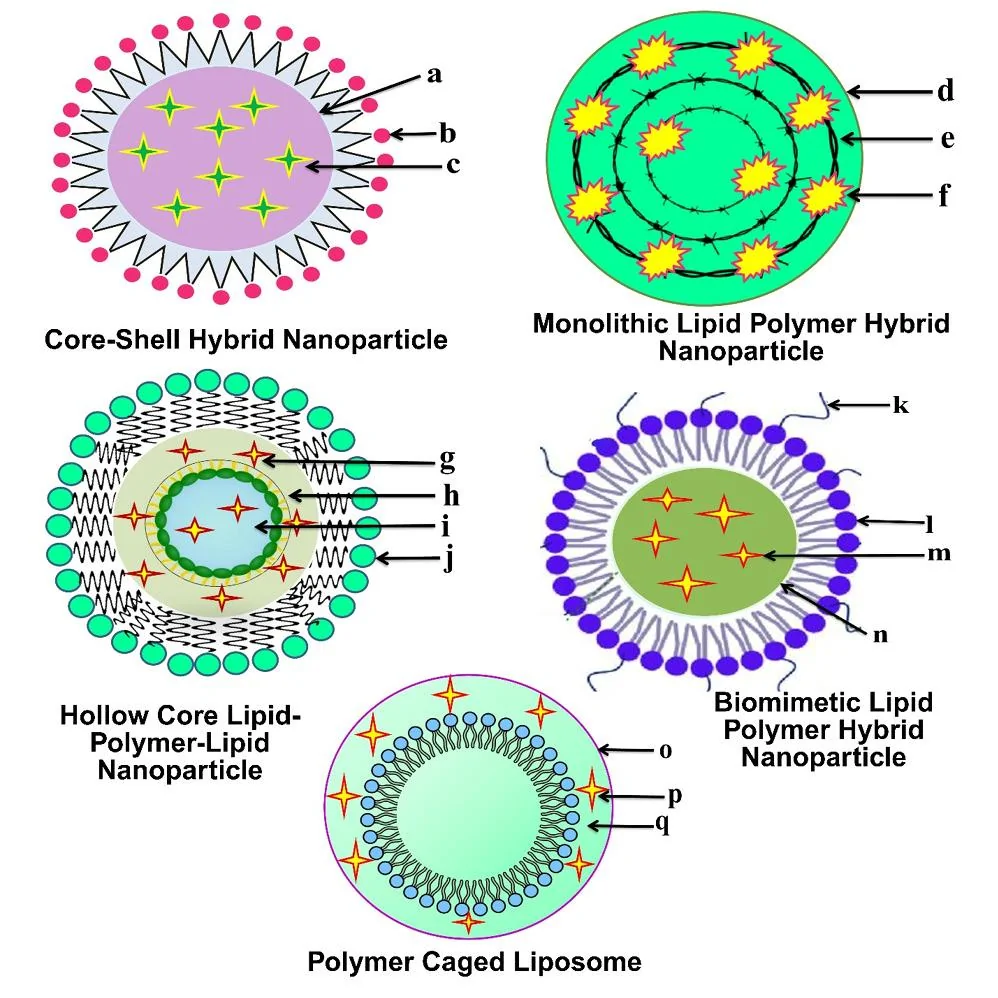
Different types of lipid-polymer hybrid nanoparticles (LPHNPs). In Core-shell hybrid nanoparticles: (a) polymer core, (b) lipid shell, (c) drug molecules dispersed within the polymer core. Monolithic lipid-polymer hybrid nanoparticles: (d) polymer matrix, (e) lipid matrix, (f) drug molecules. Hollow core lipid-polymer-lipid nanoparticles: (g) drug molecules, (h) inner lipid layer, (i) hollow core, (j) outer lipid layer. Biomimetic lipid-polymer hybrid nanoparticles: (k) lipid-PEG chains, (l) erythrocyte membrane coating, (m) drug molecules, (n) polymer core. Polymer-cages liposomes: (o) polymer coat, (p) drug molecules, (q) lipid shell

 The various LPHNP classes demonstrated a range of design approaches, each with its own therapeutic potential and drawbacks. The most basic LPHNP assemblies are monolithic ones, which combine polymers and lipids in a single matrix. Although they make formulation simple and combine the benefits of both systems, their limited structural flexibility and potential for drug leakage limit their wider clinical use. With the lipid layer stabilizing the core, polymer-core lipid-shell systems offer better biocompatibility and superior encapsulation of hydrophobic medications. Long-term stability may be compromised through, by lipid oxidation and batch variability. Through several barrier layers, L-P-L layered nanoparticles provide strong protection and improved encapsulation efficiency for labile medications. However, scalability is frequently constrained, and their complicated preparation, increased expense, and charge-dependent aggregation risk continue to be obstacles. LPHNPs coated with erythrocyte or platelet membranes are examples of biomimetic systems, which offer site-specific delivery, extended circulation, and immune evasion. Although encouraging, source variability, the difficulty of membrane isolation, and the possibility of immunogenicity if heterologous membranes are employed restrict their translation. Lastly, stimuli-responsive drug delivery is made possible by polymer-caged liposomes, which combine polymer-controlled release with liposomal versatility. Their main drawbacks are their limited large-scale reproducibility, stability in physiological conditions, and complexity of manufacturing. All things considered, even though each type exhibits advantages in a particular field, their drawbacks imply that no single design is always the best. In order to ascertain which architectures best balance stability, safety, and therapeutic efficacy, future research will probably concentrate on hybridizing these approaches (for example, applying biomimetic coatings over polymer-caged liposomes), incorporating green scalable techniques, and carrying out thorough comparative in vivo studies.

## Preparation of LPHNPs

 In general, researchers documented that there are two primary ways to prepare: single-step and double-step processes, separated into other categories. Below is a description of every preparation technique.

###  One-Step Process 

 The one-step process is one of the widely used methods for producing hybrid nanoparticles. The drug and polymer are dispersed in organic solvents and are not soluble in water. The lipids are dispersed throughout the aqueous phase by heating or sonication. The addition of the organic phase also stirs up the aqueous phase. The final product serves as the foundation for polymeric nanospheres covered in lipids. By letting it evaporate under low pressure, the organic solvent is removed. This process is quick, inexpensive, and saves time, which is why it is the most widely adopted technique for the preparation of hybrid NPs. One of the most important factors influencing drug loading, release, and entrapment efficiency is the lipid/polymer ratio.^61^ As the drug molecules diffuse out of the polymer core, the lipid coat acts as a barrier. Additionally, compared to basic polymeric nanoparticles, the lipid provides a more controlled drug release profile by sustaining the drug release for a longer duration. The single-step technique is further divided into two categories that are discussed below. Further, different drugs were formulated by using one-step methods that are presented in Table 2.

**Table 2:** Drugs formulated in LPHNPs by using one step method and two step method

**Drug**	**Disease**	**Method of preparation LPHNPs**	**Key findings**	**Critical Review/ Research gap**	**References**
Glycoalkaloids from Solanum Plant	Cutaneous Melanoma (Skin Cancer)	One-step hot-melt emulsification followed by ultrasonication / SLPHNs	Long term colloidal stability over 6 months SLPH-GE demonstrated two fold greater selective cytotoxicity toward melanoma cells compared to normal HFF-1 fibroblast cells, Enhanced skin penetration (~ 77μm)	The study lacks information on systemic toxicity and in vivo efficacy, despite demonstrating good stability and skin penetration in vitro. Unresolved issues include long-term skin safety, manufacturing scalability, and dose dependent toxicity of glycoalkloids. Whether selective in vitro cytotoxicity will result in safe and efficient tumor reduction in vivo is a significant open question.	^62^
Curdlan Mycolic acid (Immunomodulatory agents)	Immunotherapy, bacterial infection	Emulsification solvent evaporation	Zebrafish larvae used as an in vivo model Curdlan induced complex immunoregulatory responses, inflammation remained elevated Zebrafish larvae proved to be valuable model for NP based immunotherapies	Although there is limited translational value, zebrafish data demonstrate complex immune modulation; safety and efficacy in mammals are not evaluated. Uncertainty surrounds the balance between protective immunity and detrimental long-term effects, pro-inflammatory risks, and unresolved mechanisms of persistent inflammation.	^63^
Propranolol Hydrochloride	Migraine (Brain delivery)	Single emulsion/ solvent evaporation method	Higher brain accumulation after intranasal vs. oral administration Sustained release up to 8hr High radiolabeling yield 91.40 ± 1.85%	Long-term exposure, therapeutic efficacy in migraine models, and safety (nasal/olfactory toxicity) were not evaluated, despite the fact that intranasal PLHNPs exhibit early brain targeting and sustained plasma levels. Concerns about reproducibility are raised by moderate PDI, and translational relevance has not been established.	^64^
Tamoxifen citrate	Breast cancer	Ionic gelation method (one step method)	Oral controlled release of tamoxifen up to 72 hr targeting breast cancer. No toxicity on rabbit models safe interaction with blood components	Successful formulation, Invitro/limited in-vivo(rabbit) safety/ hemocompatibility, and sustained release are reported by the authors. In order to support therapeutic claims and translation, the study specifically mentions the necessity of thorough PK profiling, long term toxicity studies models. The article does not fully characterize stability under storage conditions or scale-up parameters.	^65^
Epigallocatechin-3-gallate (Green Tea)	Tooth decay	One step Emulsion and sonication method	Reduction ~15-fold in minimum inhibitory concentration (MIC) and minimum bactericidal concentration, enhanced antibacterial activity vs planktonic microbes	Although EGCG LPHNPs show good antibacterial stability and efficacy in vitro, their clinical applicability is unknown due to their lack of validation in in-vivo infection models, biofilm environments, and saliva/enzymatic conditions.	^66^
Ivermectin	Cancer (Pulmonary delivery)	Emulsion solvent evaporation	Sustained release 50-60% over 96 hrs. Pulmonary administration. Good aerosolization properties like 60 L/min flow rate. Fine particle fraction 18.53% to 24.77%	Formulation and inhalation performance; the authors and reviewers call for in vivo pulmonary distribution, local toxicity, and antitumor efficacy studies and discuss the necessity of a cry protectant during freeze-drying to avoid aggregation. Although the article highlights the feasibility of formulation, it makes clear that pulmonary safety and therapeutic index are still unknown.	^67^
Abietic Acid	Inflammation and oxidative stress	Microinjection Technique	2.49 fold increase in gut permeation, enhanced drug release, greater antioxidant and anti-inflammatory effect	No in vivo pharmacodynamics or toxicity data; gut absorption mechanisms need clarification.	^68^
Papain	Bacterial infection and enzyme deficiency disorders	Double emulsion solvent evaporation	High encapsulation efficiency, superior drug loading, enhanced antibacterial activity, excellent biocompatibility	More research is needed to determine whether papain is degraded by enzymes during processing or storage and whether it exhibits proteolytic activity in vivo. In-vivo pharmacodynamics and toxicity remain unaccessed, in-vivo validation is needed.	^69^
Gentamicin	Bacterial infection Multidrug- resistant bacterial infection	Double emulsion solvent evaporation	Upto 160-fold reduction in MIC/MBC values, stable formulation, enhanced antibacterial performance	Although preprint lacks peer reviewed validation, it exhibits strong in vitro potency. Stability, toxicity, in vivo efficacy, and formulation repeatability/scale-up have not been tested.	^70^
Ketoconazole	Superficial fungal skin infection (Topical application)	Ionic gelation technique	Enhanced antifungal activity, pH-sensitive release, reduced hepatotoxic risk	In vivo skin penetration and systemic safety not demonstrated; long-term topical tolerability unassessed.	^71^
Posaconazole	Fungal infections	Modified hot emulsification-homogenization	Sustained over 18hr with 92% release stability over 45days	Lacks in vivo pharmacokinetics and efficacy; long-term stability under physiological conditions unverified.	^46^
CRISPR-Cas9 system targeting STAT3	Hypervascularized Glioblastoma	Multicompartmentalized LPHNPs, stimuli- responsive design	Efficient targeting NPs reached glioblastoma in 2hrs responsive to tumor hypoxia ~50% STAT3 gene knockout	In vivo gene editing efficiency, immune response, and off-target effects not evaluated.	^72^
Rapamycin	Rhabdomyosarcoma Soft tissue cancer	Solvent evaporation	Induced RD and RAW (macrophage), cytotoxicity and autophagy stimulation Controlled release, improved drug stability	In vivo efficacy, toxicity and pharmacokinetics remain untested; controlled release optimization in vivo unclear.	^73^
5-Fluorouracil	Colorectal cancer	Nanoprecipitation method	EE- 80%-92% sustained release upto 72 hr, IC50 value- 2.06 fold lower in HT-29, 1.83-fold lower in HCT116	There are insufficient studies on Pharmacokinetics, systemic toxicity, invivo antitumor efficacy, and comparison with conventional 5-FU formulations; more research is required to optimize the polymer lipid ratio and avoid premature drug release.	^74^
Rivastigmine Convolvulus pluricaulis extract	Neurodegenerative diseases	Modified film hydration technique	Controlled and sustained release profile over 24hr, effective brain targeting and neuroprotection	Translation to disease models is unclear, and long term brain targeting, in vivo efficacy, and systemic safety have not been tested.	^75^
Paclitaxel, Curcumin	Breast cancer	Nanoprecipitation method	Controlled, tumor targeted release 1.6 fold higher release of Ptx at pH 5 and 1.7 fold higher release of cUR at pH 5 vs pH 7.4	No in-vivo biodistribution, PK or efficacy data available; particle heterogeneity and reproducibility need optimization; in vivo targeting/stability unverified.	^76^
Buspirone Hydrochloride	Generalized anxiety disorder	High pressure homogenization	3.29 fold increase in oral bioavailability, PEM coating using layer-by-layer technology is an effective strategy to targeted delivery	Pharmacokinetics and systemic safety have not yet been investigated; additional research is required to determine the layer-by-layer formulation’s scalability, long-term stability, and repeated-dose effects.	^77^
Posaconazole	Recurrent Vaginal Fungal infections	Emulsification followed by solvent evaporation	Sustained manner for upto 24 hr Vaginal delivery, targeted treatment of recurrent and drug resistant vaginal fungal infections. Improved tissue uptake. Minimal cytotoxicity	The author specifically suggest comparison to commercial vaginal formulations, local irritation/chronic safety studies, and prolonged in vivo infection and recurrence models. They also point out that more research is needed on mucosal retention time and formulation reproducibility.	^78^
Sorafenib	Cancer (Hepatocellular carcinoma, Renal cell carcinoma, Thyroid carcinoma)	Nanoemulsion template method	Improved solubility, controlled, sustained release, High encapsulation efficiency, Enhanced oral bioavailability	Long-term stability under physiological conditions, systemic toxicity, and in-vivo pharmacokinetics have not been tested; formulation scalability and reproducibility require additional assessment.	^53^
Paclitaxel Tamoxifen	Triple- Negative Breast cancer High recurrence, metastasis, mortality rates	Nanoprecipitation method	~31% apoptosis targeted Cytotoxicity in 3D cultures confirmed higher anti-cancer efficacy Synergistic drug action	TNBC targeting and cytotoxicity have been shown in vitro; however, aptamer immunogenicity, systemic toxicity, pharmacokinetics, and in vivo efficacy have not yet been investigated.	^79^

####  Self-Assemble Nanoprecipitation

 A significant manufacturing yield of lipid-polymer particles smaller than 100 nm was demonstrated by the self-assembled nano-precipitation technique. To create a homogenous dispersion, the polymers and medications were first dissolved in water or another solvent (organic), and then lipid or lipid polyethylene glycol was solubilized in water at about 65 to 70°C. Following constant stirring or sonication of the mixture, the polymeric drug solution was added drop by drop for precipitation of the polymer and lipid polyethylene glycol, which subsequently self-assembled with hydrophobic contacts around the polymer core. A water-miscible head was attached to the external environment, and a water-immiscible lipid tail was attached to the polymeric core to develop LPHNPs that were successfully stabilized by lipid or lipid polyethylene glycol. The LPHNPs were further centrifuged to remove excess lipids along with the polymer for evaporation of the solvent.^51^

####  Modified Emulsification-Solvent-Evaporation (ESE) 

 The sole and dual type ESE technique reported earlier was utilized in this case.^80^ In this method, lipids are mixed with water by stirring and sonication. After that, the organic solvent, polymer, and drug were dissolved by heating. The organic phase is added drop by drop to the water phase, and then probe sonication makes nanoparticles with a lipid coating that are round. After that, a rotary evaporator is used to get rid of the organic solvent, and the mixture is stirred overnight. Cold centrifugation and repeated washing clean up the suspension, and then the nanoparticles are lyophilized to make a solid form.^81^

#####  Single Emulsification-Solvent-Evaporation

 The single ESE method starts with dissolving the drug and polymer in a lipophilic solvent. This makes an O/W emulsion with a lipid water phase. Then, the solvent is allowed to evaporate. This makes LPHNPs that are more stable and have a higher yield than nanoprecipitation.^82,83^ Figure 2 represents the Single ESE method. Albash et al. have prepared propranolol HCl LPHNPs by using a single ESE method; the formulation was administered intranasal route which resulted in higher brain accumulation. With a particle size of approximately 104.5 nm, a PDI of 0.429, zeta potential of ~23.7 mV, and an EE of approximately 78%, the optimized formulation (F2) demonstrated stability over a period of 90 days. In vivo biodistribution studies were made possible by radiolabeling with ~99mTc, which produced a high labeling efficiency (~91.40%) and demonstrated sustained plasma levels for up to 8 hours, a brain: blood ratio of ~2.42 at 0.5 hours after intranasal dosing, and 5.80% ID/g brain uptake. Despite the encouraging brain targeting, the study’s in vivo assessment is restricted to short-term biodistribution at 0.5 hours and does not investigate longer term brain retention, therapeutic efficacy (for example, in neurological or migraine models), or safety (for example, histopathology, nasal mucosal irritation). Reproducibility and scale-up may be hampered by the moderate heterogeneity indicated by the comparatively high PDI (0.429). Additionally, the study ignores possible toxicity in brain tissues or the effects of repeated dosing, both of which are critical to confirming translation to clinical use.^64^

**Figure 2:**
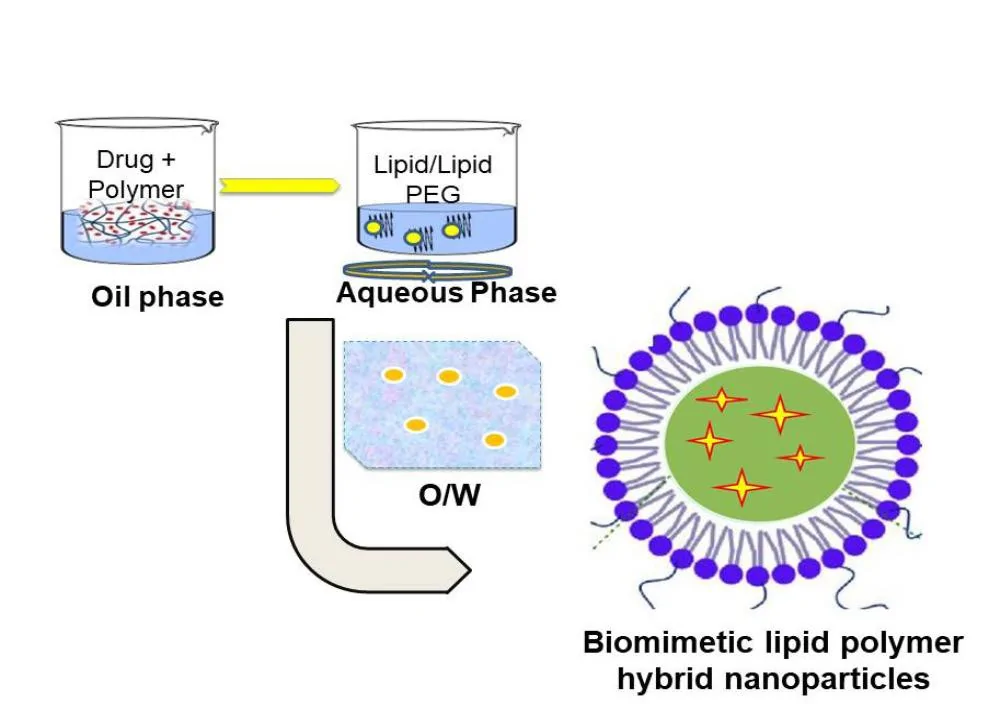
Schematic illustration of single-step emulsification solvent evaporation method

#####  Double Emulsification-Solvent-Evaporation 

 The double ESE technique was used for the hydrophilic drugs. In order to create a W/O emulsion, the drug was first mixed with water, followed by the addition of the oil phase that contains lipids and polymers. Water, lipid-PEG, and this are combined to create a W/O/W emulsion. LPHNPs are created when the solvent evaporates. They have a unique structure consisting of an aqueous core that is coated with lipids, a polymer layer surrounding it, and an external lipid-PEG shell.^84^ Figure 3 represents the double ESE method. Yassin, in 2025, using a double emulsion solvent evaporation technique, formulated gentamicin-loaded LPHNPs to treat bacterial infection and multidrug-resistant bacterial infection.^76^

**Figure 3:**
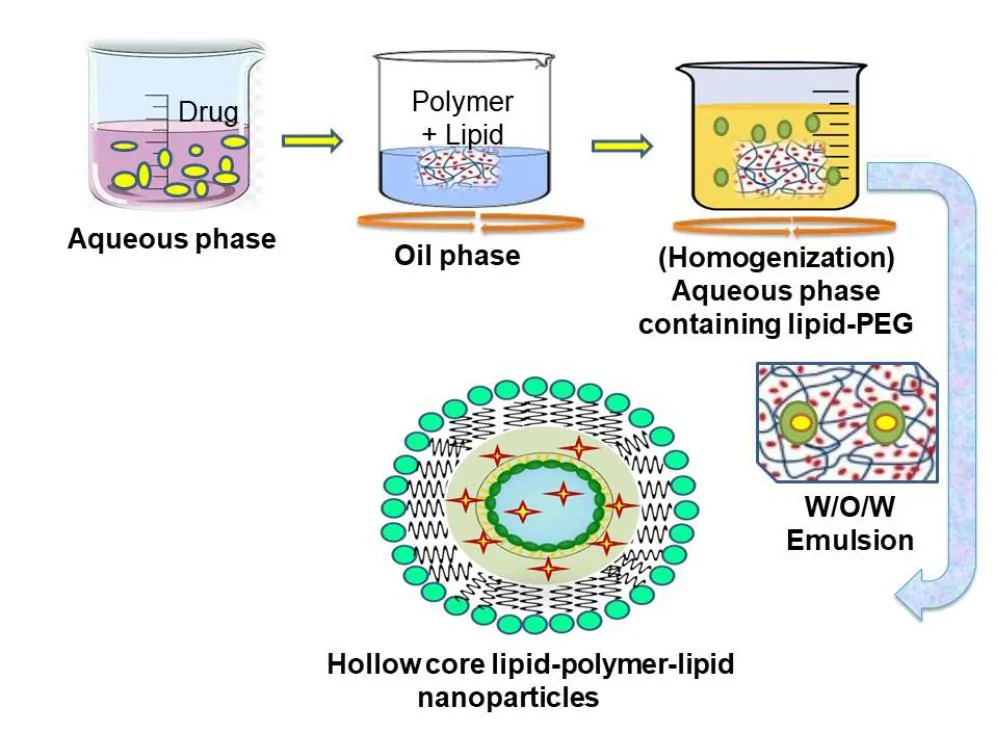
Schematic illustration of double emulsion solvent evaporation

###  Two-Step Method 

 In a two-step method, which is frequently employed to create hybrid nanoparticles, lipid vesicles and polymeric cores are synthesized independently. These components are then combined using methods like sonication, extrusion, or microfluidics to create LPHNPs. This multilayer approach improves formulation stability, prolongs release, reduces premature drug leakage, and boosts drug encapsulation effectiveness.^85^ Different drugs like posaconazole, rapamycin, paclitaxel, etc. were formulated by using a two-step method, the details are given in Table 2.

####  Conventional Two-Step Method

#####  Emulsification-Solvent-Evaporation 

 The traditional two-step emulsification-solvent-evaporation method is frequently used to manufacture LPHNPs. By dissolving a drug molecule and a hydrophobic polymer, such as PLGA, in an organic solvent, this method develops an oil phase. W/O emulsion, which is the primary emulsion, is then created by emulsifying the oil phase with an aqueous phase. This is re-emulsified into a second aqueous phase containing a stabilizer like polyvinyl alcohol (PVA) to produce a W/O/W double emulsion. When the solvent evaporates, solid NPs with a polymeric core and lipid shell are produced. This method boosts drug encapsulation, promotes controlled release, and increases stability.^86^ In 2025, posaconazole-loaded LPHNPs were demonstrated by Deshkar et al. with an average particle size of 465 ± 58 nm, a PDI of 0.17 ± 0.07, a zeta potential of -12 mV, and a high EE (~91%), resulting in enhanced cellular uptake and sustained drug release over a 24 hour period with minimal cytotoxicity. Although the technique offers superior drug encapsulation and controlled release, there are still issues with the internal aqueous core’s scalability, reproducibility, and structural stability. Its wider translational application is limited by variations in particle size and encapsulation efficiency at larger scales, the possibility of early drug leakage, and sensitivity to formulation parameters (solvent removal, homogenization, and surfactant concentration). To improve industrial applicability and therapeutic consistency, future research should concentrate on standardizing process parameters, enhancing nanoparticle core stability, and investigating alternative hydrophilic drug encapsulation techniques (such as electrohydrodynamic atomization or microfluidics).^78^

#####  Nanoprecipitation

 The nanoprecipitation method, which involves dissolving the polymer and drug in a water-soluble organic solvent, is used to create LPHNPs. The solvent is added then drop by drop to an aqueous lipid dispersion that is kept above the gel-to-sol transition temperature of the lipid. Lipids self-assemble around the developing polymeric core to form a stable hybrid structure, while solvent diffusion causes fast polymer precipitation. Particle size is further reduced using sonication or homogenization. The lipid/polymer ratio critically influences nanoparticle formation; excessive lipids can form liposomes, while insufficient lipids may lead to aggregation. The incorporation of PEGylated lipids enhances colloidal stability via steric hindrance, preventing aggregation without compromising drug loading or release efficiency.^87,88^ Figure 4 represents the nanoprecipitation method. Paclitaxel,^76^ Tamoxifen,^79^ and 5-Fluorouracil^74^ were formulated by using the nanoprecipitation method; the details are given in Table 2.

**Figure 4:**
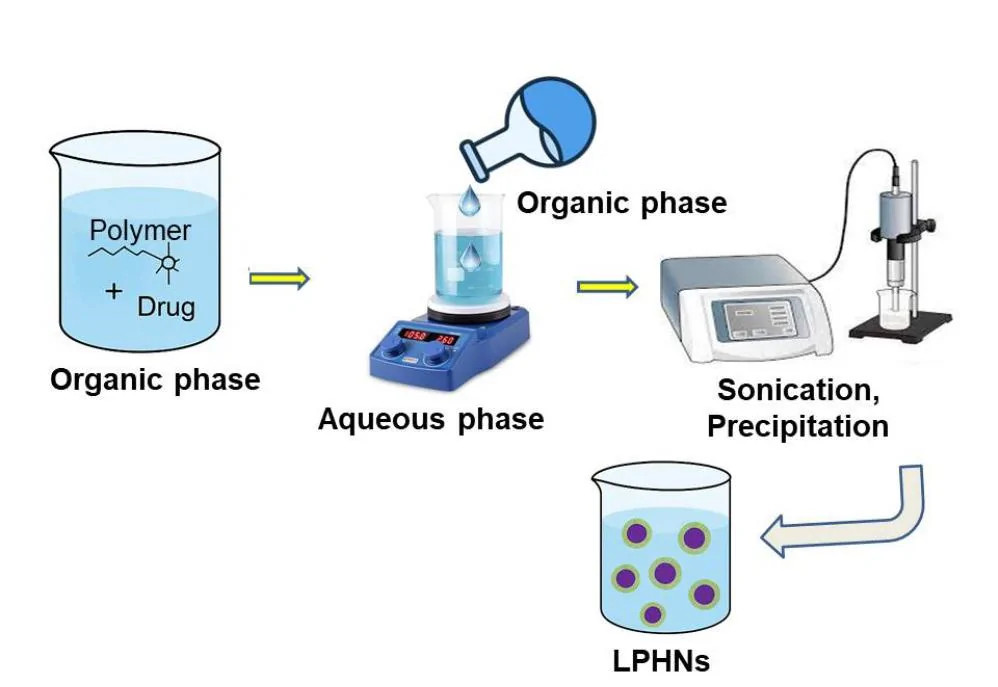
Schematic illustration of nanoprecipitation

#####  High-Pressure-Homogenization 

 High-pressure homogenization (HPH) is a widely employed procedure for preparing lipid-polymer hybrid nanoparticles (LPHNPs), offering advantages such as scalability, solvent-free processing, and efficient encapsulation. This method involves dispersing a lipid phase containing the drug in an aqueous surfactant solution, followed by homogenization under high pressure (typically between 100 and 2000 bar) through a narrow orifice. The resultant shear forces and cavitation break down the lipid droplets to nanoscale dimensions, forming a stable nanoparticle dispersion. HPH can be achieved using either cold or hot homogenization conducted above the lipid’s melting point, while cold homogenization is suitable for heat-sensitive compounds. The technique has been successfully applied in the preparation of various LPHNPs, demonstrating its versatility and effectiveness in nanocarrier development.^89,90^ Dangre et al. 2024 developed BuspironeHCl-loaded LPHNPs by using the above method, which results in 3.29-fold increase in oral bioavailability.^77^

####  Non-Conventional Two-Step Method 

 Larger-scale LPHNP production is the primary application for the non-conventional approach. To prepare LPHNPs, this technique additionally uses soft lithography, particle molding, and spray drying.

#####  Spray-Drying 

 Polymeric nanoparticles, averaging 400-500 nanometers in size, were generated using the spray drying method and subsequently dispersed in dichloromethane having lipid components. For producing “Lipid-Polymer Hybrid Nanoparticles” of about 0.9 to 1.2 micrometers, this dispersion was subjected to another round of spray drying using a spray drier that was not appropriate for NP production.^84^

#####  Soft Lithography

 Recently, the production of LPHNPs for the conveyance of inherited materials has been investigated using a method for molding soft lithography particles called particle replication in non-wetting templates.^91^

 While both one-step and two-step methods for preparing LPHNP have unique benefits, they also have serious drawbacks. Early-stage proof-of-concept studies benefit greatly from the one-step process’s simplicity, speed, and affordability. Its moderate polydispersity, residual solvent issues, and high sensitivity to formulation parameters, however, restrict reproducibility and make scale-up difficult. The two-step process, on the other hand, overcomes some of the shortcomings of one-step methods by offering better control over particle architecture, increased encapsulation efficiency, and improved long-term stability, despite being more labor-intensive and equipment-dependent. The two-step category’s high-pressure homogenization provides a more translational pathway while also addressing scalability and solvent toxicity concerns. However, there are certain issues that both approaches have in common, such as a lack of long term in vivo safety data, complexity in scaling up, and incomplete standardization. Achieving repeatable, clinically viable LPHNP formulations in the future will depend on the combination of solvent free and green manufacturing techniques, the use of scalable methods like microfluidics and HPH, and methodical comparison between approaches.

## Characterization

###  Surface Morphology 

 In general, advanced imaging techniques such as atomic force microscope (AFM), TEM, as well as SEM can be employed to characterize the exterior morphology and structure of LPHNPs. These techniques provide overall morphology, surface roughness, and the shape of the particles. Zhang et al. have demonstrated that TEM and particularly negative staining TEM are a valuable method in the study of the corona and the core-shell structure of LPHNPs.^92^

###  Particle Size and Distribution 

 DLS (Dynamic light scattering), which offers precise and fast observations, is commonly used to assess the PDI (Polydispersity index) and particle size of LPHNPs, which are significant factors that affect their biological behavior, such as cellular absorption and circulation time. Monodisperse LPHNPs, which are typically between 10 to 100 nm in size, are considered to be the best for intravenous administration. Notably, Valencia et al. used a microchannel-based nanoprecipitation methodology to successfully manufacture LPHNPs with an average diameter of about 40 nm, demonstrating good size uniformity.^93^

###  4.3. Zeta Potential 

 ZP is the surface charge of the nanoparticles, which is measured by using laser Doppler velocimetry and photon correlation spectroscopy or by using instruments like Malvern Zetasizer.^94^

###  Drug-Loading and Encapsulation-Efficiency

 EE and DL capacity can be measured by using the following formula ^95^


$$\begin{array}{l} Encapsulation\;Efficiency\left(\%\right)=\left(\frac{Amount\;of\;drug\;added-Amount\;of\;drug\;in\;supernatant}{Amount\;of\;drug\;added}\right)\times 100\\ Drug\;loading\left(\%\right)=\left(\frac{Amount\;of\;entrapped\;drug\left(\mu g\right)}{Amount\;of\;entrapped\;drug\left(\mu g\right)}\right)\times 100 \end{array}$$

###  In-Vitro Drug Release Studies 

 The dialysis method is the most commonly used analytical technique for evaluating in vitro drug release profiles, and it is frequently combined with UV-visible spectrophotometry or high-performance liquid chromatography(HPLC) for quantification. The drug release behavior of LPHNPs is a crucial parameter for assessing their therapeutic potential and controlled release capabilities.^96^

###  In-Vivo Release Studies 

 In vivo evaluation is essential for verifying the safety, pharmacokinetics, biodistribution, and therapeutic effectiveness of LPHNPs, which have shown reduced off-target toxicity, augmented tumor accretion by the enhanced permeability retention effect, and extended circulation times in a variety of animal models. Wong et al. showed that PEGylated LPHNPs significantly improved bioavailability and decreased hepatic clearance, while Zhang et al. (2008) reported that LPHNPs loaded with docetaxel showed superior tumor growth inhibition and lower systemic toxicity compared to free drug and conventional formulations. These findings highlight the translational potential of LPHNPs as multi-functional platforms for targeted drug delivery as well as controlled-release in vivo.^92^

###  Stability Studies 

 Determining the integrity, along with the shelf life of LPHNPs under varied physiological and storage environments, requires a stability study. LPHNP structure combines a polymeric core with a lipid or PEG lipid shell, which ensures improved colloidal stability by decreasing aggregation and halting premature drug leakage. Valencia et al. in 2010 reported that LPHNPs exhibited good physical stability over a period of weeks when stored at 4 degrees centigrade, maintaining consistent particle size and drug encapsulation efficiency.^93^ Additionally, it has been demonstrated that PEGylation of the lipid layer increases resistance to enzymatic degradation and serum protein adsorption, increasing the durability of nanoparticles in biological conditions.^92^ LPHNPs could be proven to be a good candidate for systemic delivery and long-term preservation because of these qualities.

## Factors Affecting Hybrid Nanoparticles

 The structural and functional properties of LPHNPs are profoundly influenced by several formulation variables. Lipid coating plays a critical role in modulating drug release, acting as a diffusion barrier that enhances encapsulation efficiency by minimizing premature drug leakage. The lipid-to-polymer ratio is another key parameter that directly affects particle size, morphology, and the integrity of the lipid shell. Optimal ratios, such as 15% (w/w) for PLGA-lecithin systems, ensure stable hybrid structures, while excessive lipid content may result in liposome formation. The nature of the polymer, including its density, charge, and compatibility with lipid components, also governs nanoparticle stability, zeta potential, and drug release behavior. For example, anionic polymers like PLGA interact favorably with cationic lipids to form stable complexes. Additionally, the incorporation of PEG provides steric stabilization at physiological pH and prevents aggregation, with a 25% lipid-to-PEG ratio found to yield nanoparticles with optimal size and dispersion. Collectively, these formulation elements must be finely tuned to achieve the desired therapeutic and physicochemical profile of LPHNPs.^24^

## Applications of LPHNPs

###  Drug Delivery System 

 LPHNPs offer a robust platform for the delivery of hydrophilic and hydrophobic drugs together. Their core-shell structure, combining a polymeric core and a lipid shell, enhances drug encapsulation efficiency and stability. For instance, multi-layered polymeric core lipid hybrid nanoparticles(PCLHNPs) have been utilized to co-deliver chemotherapeutic agents like oxaliplatin, camptothecin, and 5-fluorouracil, demonstrating controlled release profiles and improved therapeutic efficacy.^58,97^

###  Cancer Therapy 

 LPHNPs are particularly effective in targeted cancer therapy. Functionalization with ligands such as folate or transferrin allows for selective distribution to cancer cells, reducing off-target impacts. For example, hybrid nanoparticles functionalized with folic acid and loaded with gefitinib and yttrium-90 exhibited enhanced tumor accumulation and synergistic chemoradiotherapy effects in nasopharyngeal cancer models.^58^ Drugs like Cisplatin,^72^ doxorubicin,^35^ olaparib,^98^ and many more drugs were formulated as LPHNPs for effective cancer cell targeting drug delivery; the details are given in Table 3.

**Table 3:** LPHNPs formulation for cancer therapy

**Name of the Drug**	**Therapeutic category**	**Area of Application**	**Ref.**
Paclitaxel (PTX) and tributyrin (TB)	Antineoplastic agent Histone deacetylase (HDAC) inhibitor	Drug is used to treat Colorectal cancer (CRC), multiple parameters optimized like surface mean size 1000 nm, EE: 99.9 ± 0.2 % with production yield: 97.2 ± 0.08 %, cytotoxicity IC50 to 83.7nM to 199.5nM.	^99^
Resveratrol (RSV)	Glioblastoma cancer cell	An uptake of 162.26% for “resveratrol loaded lipid polymer hybrid nanocarriers” was observed in a cellular uptake studies using U87 cells compared to reference (99.38 %), representing extreme cellular internalization. Different dosed of resveratrol exhibited a greater percentage of inhibition along with anticancer activity against gliobastoma cancer cell line rather than 5-FU. The study confirms the probability of “resveratrol loaded lipid polymer hybrid nanocarriers” for the efficient control of glioblastoma multiforme.	^100^
Cisplatin (CDDP) and afatinib (AFT)	Nasopharyngeal carcinoma (NPC)	Cisplatin and anethole combinedly exhibited improved anticancer activity in a 1:1 ratio. While Cisplatin alone showed 85.1% cell viability, compared to 39.5% using CDDP+AFT combination. Cisplatin and anethole containing LPHN displayed greater cytotoxicity than the free drug cocktail, confirming the enhanced efficacy of the nanoparticle system in reducing viability, inducing programmed cell death, arresting cell movement, cell cycle and cell division.	^101^
Doxorubicin (DOX)	Antineoplastic & anthracycline agent	DOX-loaded LPHNs:121.90 ± 3.0 nm,Drug loading: 0.391% ± 0.01,(EE):95.5% ± 1.39,Bioavailability (AUC):~2-fold increase	^41^
Gemicitabine and HIF-1 alpha SiRNA (combo)	Anticancer & Antineoplastic	LENP-Gem-siHIF1α treatmentinhibited tumor metastasis in anorthotopic pancreatic cancer model, HIF1α expressionin bothin vitro and in vivo	^102^
Gemcitabine Hydrochloride (GEM)	Antineoplastic /anticancer/anti-tumor agent	Cytotoxicity of GEM-loaded LPHNs was tested using MTT assays. The tests were done on MCF-7 and MDA-MB-231 breast cancer cells. Pharmacokinetics and in vivo anticancer studies were done in rats. Female Sprague-Dawley rats received the drug by intraperitoneal injection. The GEM-loaded LPHNs showed better results than the commercial drug Gemko®. In rats, the GEM-loaded LPHNs stayed longer in the bloodstream. The half-life of GEM in LPHNs was 4.2 times longer than native GEM. These results suggest GEM-loaded LPHNs may improve breast cancer treatment.	^103^
Olaparib	Poly(ADP-ribose) polymerase inhibitor	The Olaparib-loaded lipo-polymeric hybrid nanoparticles (OLA-LPHNs, St@OLA-LPHNs, and t/Biotin@OLA-LPHNs) had a particle size < 150 nm and zeta potential < +30 mV. They showed controlled drug release compared to free Olaparib and enhanced in-vitro cytotoxicity, with a 7.6-fold improvement in IC50 in 4T1 cells using St/Biotin@OLA-LPHNs.	^104^
Docetaxel (DTX)	Antineoplastic agent	DTX-LPHNS, FA-targeted pH-sensitive polymer-lipid nanoparticles (FA/PBAE/DTX-NPs) showed enhanced cellular uptake, lysosomal escape, and cytotoxicity in breast cancer cells. They exhibited strong tumor-targeting ability and superior antitumor efficacy with minimal systemic toxicity in 4T1 tumor-bearing mice.	^98^
5-fluorouracil	Anticancer	5-FU-loaded lipid-PLGA hybrid nanoparticles (5-FU-LPHNs) showed nano-size (155.7–316.4 nm), high entrapment efficiency (80–92%), and sustained biphasic drug release up to 72 hours. In-vitro studies confirmed significantly higher cytotoxicity in HT-29 and HCT116 colorectal cancer cells compared to free 5-FU, with ~2-fold lower IC₅₀ values, indicating enhanced anticancer efficacy.	^74^
Docetaxelpro(DTXp) and Cisplantin (DDP)	Lungs Cancer	Aptamer-decorated, Docetaxel prodrug and Cisplatin “Lipid Polymer Hybrid Nanoparticles” demonstrated enhanced cytotoxicity (IC₅₀ = 0.71 ± 0.09 μg/mL), strong synergistic effect (CI = 0.62), and high tumor inhibition (81.4%) compared to non-aptamer or single drug LPHNs.	^105^
Cisplatin (CIS) andFluoropyrimidine (5-FU)	Anticancer	In this study, TAB-CIS/5-FU LPHNs showed a mean particle size of ~100 nm and high encapsulation efficiency (~90%). They exhibited sustained drug release and achieved 63.9% cellular uptake. The in vivo model demonstrated superior antitumor efficacy. The best synergistic effect was observed at a 1:1 drug ratio, with a combination index (CI) of 0.68, indicating strong synergy.	^106^
Doxorubicin (DOX) and gallicacid (GA)	Anticancer	Hyaluronic acid modified doxorubicin and gallic acid “Lipid Polymer Hybrid Nanoparticles” exhibited noticeable cytotoxicity, and greatest synergistic action was observed with a ratio of 2:1 (doxorubicin and gallic acid). Biological studies exposed that Hyaluronic acid modified doxorubicin and gallic acid LPHNs decayed tumor growth 77.7% (956 to 213 mm^3^).	^107^
Dacarbazine	Anticancer	Dacarbazine-loaded lipid polymer hybrid nanoparticles (LPHNs) were developed for topical delivery to treat skin melanoma, aiming to reduce systemic side effects and improve skin permeability. Particle size: 202.7 nm, Entrapment efficiency: 70.29 ± 0.97%, Zeta potential: −24.89 mV.The optimized LPHNs were incorporated into a gel and tested for ex vivo permeability, showing enhanced skin permeation following Higuchi diffusion kinetics, with a flux of 15.93 ± 1.61 μg·cm⁻^2^·h⁻¹. In vitro cytotoxicity on melanoma cell lines showed improved anticancer activity.	^108^

###  Gene and mRNA Delivery 

 As efficient carriers for the transport of genes and mRNA, LPHNPs provide defense against enzymatic degradation and promote cellular absorption. LPHNPs are appropriate for gene therapy applications due to their capacity to encapsulate nucleic acids and release them in a regulated fashion.^109^ By combining siRNA-loaded lipid-polymer hybrid nanoparticles with focused ultrasound (FUS), Yang et al. (2021) described a novel approach to treating drug resistant glioblastoma that temporarily disrupts the BBB. By addressing two significant challenges in glioblastoma therapy BBB impermeability and chemotherapy resistance this combinatorial approach not only increased brain penetration but also improved gene silencing efficacy. According to the study, FUS offered spatially controlled BBB modulation, which led to better therapeutic results, while LPHNPs offered stability, biocompatibility, and effective gene loading. The work has some limitations in spite of its novelty. Particularly in chronic treatment settings, the safety and reversibility of repeated FUS-mediated disruption of BBB are still unknown, and the long term immunological effects of repeated exposure to siRNA-LPHNP have not been thoroughly assessed. Furthermore, although the proof-of-concept in animal models is convincing, large-scale nanoparticle synthesis under repeatable conditions, dosing optimization, and ultrasound parameter optimization are necessary for translation into clinical practice. Overall, the study presents encouraging opportunities for synergistic nanomedicine-device combinations in the treatment of glioblastoma; however, prior to practical implementation, additional validation in sophisticated preclinical and clinical models is essential.^110^

###  Diagnostic Imaging 

 By encasing them inside the polymer core, the LPHNPs can also be utilized as delivery vehicles for bio-imaging agents for medical diagnostics, such as iron oxide, fluorescent dyes, quantum dots (QDs), and for computed tomography (CT) and magnetic resonance imaging (MRI), Inorganic nanocrystals are frequently used.^111^ By adding gold nanocrystals and quantum dots, Mieszawska et al. (2012) presented a novel method for engineering LPHNPs that could provide both drug delivery and imaging capabilities. This approach demonstrated how versatile LPHNPs are as delivery and theranostic agents. However, their research was restricted to macrophage studies conducted in vitro: there was no confirmation of biodistribution, toxicity, or imaging in vivo. Concerns about the long-term stability, potential cytotoxicity of quantum dots, and conversion of such hybrid nanoprobes into clinically safe systems are also still unresolved. To fully achieve theranostic potential, future studies must concentrate on biocompatibility, imaging sensitivity in intricate biological systems, and integration of therapeutic payloads.^112^

###  Stimuli-Responsive Drug Delivery System 

 LPHNPs were developed in a manner that releases the medication at the intended location and can respond to specific stimuli such as pH, temperature, as well as redox conditions. As an example, by releasing the medication in response to the tumor’s microenvironment, redox-sensitive LPHNPs functionalized with transferrin showed enhanced therapeutic efficacy against non-small cell lung cancer.^113^ By developing LPHNPs that are co-loaded with the chemotherapeutic medication camptothecin (CPT) and superparamagnetic iron oxide (FeO4) nanoparticles on a single substrate, SD Kong et al. introduce a novel stimuli-responsive drug delivery method. Excellent colloidal stability and prolonged drug release under physiological conditions are guaranteed by the lipid-polymer hybrid design. Notably, the addition of FeO4 allows for remote radio-frequency (RF) magnetic field stimulation, which heats the magnetic core and relaxes the polymer matrix, resulting in rapid CPT release and on-demand drug release. This externally adjustable, magnetically driven release method significantly improves the precision and controllability of chemotherapy delivery by increasing cytotoxicity against cancer cells while reducing systemic exposure. In vitro controlled release and enhanced cytotoxicity were demonstrated by RF-activated LPHNPs, but their translational potential is limited by the lack of in vivo efficacy, biodistribution, and safety data as well as unresolved problems with heating uniformity and scale-up reproducibility.^114^

###  Vaccine Delivery

 In vaccine delivery, biodegradable nanoparticles have a number of advantages, including reduced immunogenicity, decreased damage, no viral recombination, as well as lower production costs because they are stable and long-lived. Different studies show that LPHNPs are good carriers for both protein-based and nucleic acid-based vaccines, due to their ability to encapsulate antigens and adjuvants, which allows for the development of efficient vaccine formulation.^50^ Hu et al. (2014) provided a noteworthy illustration of how LPHNPs with a lipid shell and PLGA core may effectively transport the model antigen keyhole limpet hemocyanin (KLH) to dendritic cells. The work demonstrated in a novel way that the shell’s lipid composition, particularly the addition of cholesterol and cationic lipids, was crucial for increasing antigen absorption, regulating release kinetics, and boosting colloidal stability. This work provides a design-driven method for creating LPHNP-based vaccine platforms with enhanced immunogenicity and delivery efficiency, highlighting the significance of lipid selection in enhancing nanoparticle-immune cell interactions. Nevertheless, the study was limited to in vitro tests and did not evaluate safety, biodistribution, or immunogenicity in vivo. Furthermore, long-term stability was only partially assessed under static conditions, and although higher positive charge enhanced uptake, the possible trade-off with cytotoxicity and undesired immune activation was not discussed. In-vivo validation (immune response, safety, biodistribution), assessment of the cytotoxicity of high surface charge formulations, and investigation of long-term stability/release under physiologically like conditions are all necessary for clinical translation.^115^

###  Brain Delivery 

 Delivery of LPHNPs across the blood-brain barrier (BBB) has been reconnoitered. Various neurological disorders like Alzheimer’s disease and glioblastoma, which affect the BBB, can be effectively targeted by the LPHNPs.^116^ Sekerdag et al. (2017) used intranasal delivery of lipid-PEG-PLGA hybrid nanoparticles (LPHNPs) loaded with farnesylthiosalicylic acid (FTA) to present a novel treatment strategy for drug-resistant glioblastoma. Their research clearly showed that this non-invasive approach produced noteworthy therapeutic results, lowering the volume of glioma tumors in rats by 55.7%. This was equivalent to intravenous administration, but it also had the benefit of not causing systemic toxicity. This study offers strong proof of LPHNPs ability to get around the BBB, which is a major drawback of traditional glioblastoma treatment. The study is limited, though, by its reliance on small-animal models, which might not accurately capture the intricacy of human glioblastoma and nasal physiology, in spite of these encouraging results. Furthermore, there is still a lack of attention paid to the nose-to-brain transport’s reproducibility, mucosal irritation, and long-term safety. Crucially, only a portion of the mechanistic insights into the pathways by which nanoparticles are absorbed through the nasal mucosa and distributed throughout the various brain regions were investigated. To increase the clinical viability of intranasal LPHNP delivery systems for glioblastoma, future research should concentrate on converting this strategy into clinically relevant models, refining formulations for scalability, and establishing pharmacokinetic-pharmacodynamic correlations.^117^ Further resveratrol,^100^ is also formulated for glioblastoma cancer cells; the details are given in Table 3.

###  Phytochemical Delivery

 In the case of phytochemical delivery or natural source extract delivery, LPHNPs are a promising carrier, which protects the phytochemical from the outer environment and protects the drug from degradation by encapsulating it within a hybrid matrix and enhancing its therapeutic effects.^113,118,119^ In order to improve oral delivery, mucoadhesion, and pharmacological efficacy against severe acute pancreatitis (SAP), the flavonolfisetin (FST) was formulated into chitosan-coated PLGA-lipid nanoparticles in the study by Awadeen et al. (2023), which presents a novel phytopharmaceutical application of LPHNPs. This work is revolutionary because it shows, for the first time, that encapsulating FST within LPHNPs yields a sustained release profile, optimizes particle size (~125 nm), achieves high entrapment (~62%), and greatly increases its water solubility. Furthermore, oral pre-treatment with FST-loaded LPHNPs significantly reduced SAP and related multi-organ damage in rats, outperforming both free FST and empty nanoparticles, according to their in vivo evaluation. The scalability of the formulation, the possible variability of plant-derived bioactives, and the long-term biosafety of polymer-lipid-chitosan composites are still issues, even though this study makes a significant advancement by connecting LPHNPs to phytopharmaceutical nanotherapy for inflammatory disorders. To fully realize their potential in phytomedicine, future research should concentrate on evaluations of LPHNP-based combination therapies, clinical translation through standardized manufacturing, and comparative studies with other natural product loaded nanocarriers.^34^ further details are given in Table 1.

###  Combination Therapy 

 Several medications have been appropriately integrated into the matrix core-shell structure of LPHNPs. Co-administration of chemotherapeutic drugs, gene therapies, and immunomodulatory drugs has proven especially advantageous in the treatment of cancer since it can overcome drug resistance and produce synergistic results.^58^ Figure 5 represents the graphical application of the LPHNPs.

**Figure 5:**
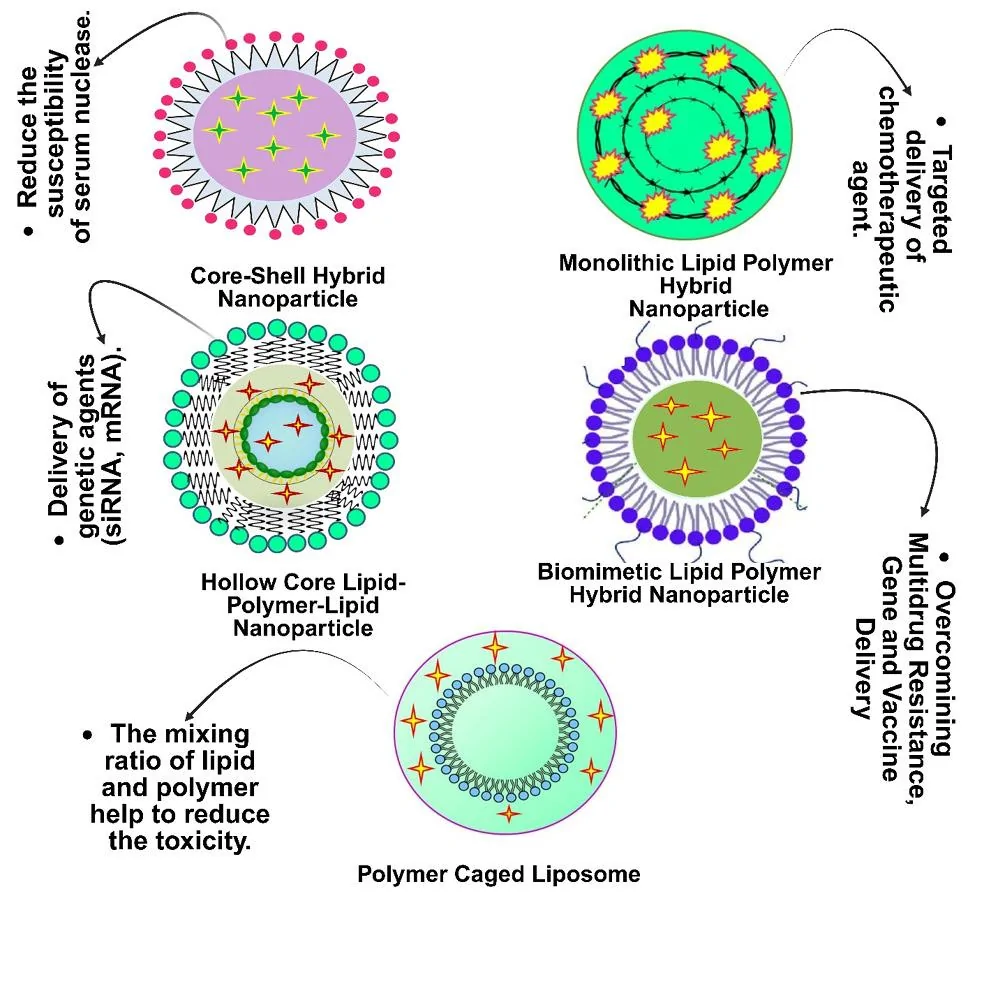
Different application of LPHNPs

## Patents of LPHNPs

 This review article highlights significant breakthroughs and technological developments in the subject by compiling an extensive list of patents published in recent years. These patents include various advancements, from innovative materials and manufacturing techniques. Further, the patent details are mentioned in Table 4.

**Table 4:** Patents of the LPHNPs system

**Author/Owner**	**Application Number/ Patent No.**	**Priority/Grant Date**	**The Title of Invention**	**Applications**	**Ref.**
YM Kim et.al	17/228224	12/09/ 2021	Dual-targeting lipid-polymer hybrid nanoparticles	Specifically the invention involves 1.dual-targeting lipid-polymer hybrid nanoparticles (T-hNPs) 2. have a polymer core containing a hemeoxygenase 1 (HO-1) inhibitor (a compound known to affect cancer progression) 3. have a lipid shell with a targeting moiety (to direct the nanoparticles specifically to cancer cells or tumor environments)	^120^
D Chitkara et.al	17/284155	12/02/2021	A lipid-polymer hybrid nanoparticle	This invention claims LPH nanoparticles comprising solid lipid, liquid lipid and amphiphilic polymer, the core contains mention polymers, while shell comprises the hydrophilic portion of the polymer.	^121^
HU Jiaming et. al,	15/831998	06/28/ 2018	Lipid-polymer Hybrid Nanoparticle Biochip and Application Thereof	Claim include a lipid polymer hybrid nanoparticle comprising: a polymer core surrounding a hydrophobic drug molecule and a lipid shell surrounding the polymer core. LPHNS particle size range of 50 nm to 200nm.	^122^
J Luo et.al	15/464918	09/21/ 2017	Lipidic compound-telodendrimer hybrid nanoparticles and methods of making and uses thereof	Patent claim on composition, Encapsulation capability, manufacturing methods and use in therapy.	^123^
CaPrestidge et. al	10,463,626	11/05/ 2019	Drug delivery composition comprising polymer-lipid hybrid microparticles	The claim specifying the active substsnce as a poorly water soluble drug, polymeric nanoparticles comprising PLGA, lipid droplets comprising MCT, the nanoparticle stabilizer being PVA or DMAB, and the microparticle being spray-dried with an average diameter under 5µm.	^124^
JO Nagy et.al	11,337,924	04/21/ 2022	Targeted polymerized nanoparticles for cancer treatment	The patent on targeted polymerized nanoparticles for cancer treatment introduces polymer-based nanoparticles that are chemically engineered for targeted delivery of anticancer drugs. These nanoparticles are surface-modified with ligands to specifically bind to cancer cell markers, ensuring precise delivery of therapeutics to tumors while minimizing toxicity to healthy tissues. The core innovation lies their controlled polymerization, enhanced stability and targeted drug release.	^125^
Z Cheng et.al	16/137155	03/28/ 2019	Polymer-lipid hybrid nanoparticles of capecitabine utilizing micromixing and capecitabineamphiphilic properties	The patent key innovative points are 1. Use of capecitabine’samphiphilic properties to improve encapsulation within the hybrid nanoparticles.2. Application of a micromixing-based method toachive uniform particle formation.3. Formulation designed to enhance bioavailability, stability, and targeted delivery of capecitabine in cancer treatment.	^126^
RJ Lee et.al	10,307,490	06/04/ 2019	Lipid nanoparticle compositions for antisense oligonucleotides delivery	This patent presents a novel LCAN platform combining albumin-polycation with lipid shell and surface targeting ligands, designed to encapsulation specific ASOs (against Akt-1 or Hif-1α) offering tunable size(≤ 150nm) robust targeting and therapeutic potential against cancer- alongside detailed formulation ratios and scalable manufacturing method.	^127^
H Gao et.al	11/848484	05/01/ 2008	Hybrid lipid-polymer nanoparticulate delivery composition	The claim of innovation: a hybrid lipid- polymer nanoparticulate delivery system comprising a biodegradable polymeric core (PLGA) encapsulating an active pharmaceutical ingredient (API), envolved by alipid shell (lecithin/DSPE-PEG2000) demonstrating enhanced bioavaibility (upto 3.2 folds), controlled release over 72 hours and improved cellular uptake (65% higher) compared to conventional polymeric or liposomal systems alone.	^128^
My jeon.et.al.	18/978701	04/03/ 2025	Method for synthesizing hybrid nanoparticles comprising apolipoproteins	The innovation lies in a one-step microfluidic method that forms lipid-coated, Apo lipoprotein-functionalized nanoparticles by precise mixing of core, lipid, and protein streams to generate uniform, bio-functional hybrid particles	^129^

## Future Perspective

 Future research efforts with respect to the use of PLGA-based lipid-polymer hybrid nanoparticles (LPHNPs) should include work on developing methods that can enhance the consistency and scalability of the formulation process. The use of solventless and scalable techniques such as high-pressure homogenization and microfluidics might help achieve this goal. Additional improvements are anticipated through surface functionalization and stimuli responsiveness approaches for enhanced site-specific and controlled drug delivery. Other applications include brain targeted drug delivery, gene and mRNA delivery, vaccine formulation, phytoconstituent delivery, and combinatorial therapies using LPHNPs. Emphasis must be placed on theranostic LPHNPs that combine therapy and diagnosis purposes since this field is relatively less explored. Pharmacokinetics, biodistribution, toxicity, and efficacy studies will be indispensable for future clinical use.

## Conclusion

 PLGA-derived LPHNPs stand out as a multifunctional nanocarrier system combining the structural benefits of biodegradable polymers along with the biological and functional characteristics of lipids. The versatility of this nanosystem can lead to better drug entrapment, sustained release, stabilization, and delivery targeting. In this review, the versatility of LPHNPs in different applications is highlighted, including conventional drug delivery systems, cancer treatment, genetic/mRNA delivery, brain delivery, vaccine delivery, diagnostics, and phytochemical delivery. However, there are still certain disadvantages associated with the complicated nature of formulations, difficulty of replicating results, scale-up problems, instability, toxicity, and lack of in vivo long-term validation. In general, LPHNPs offer a very promising tool for development of new systems for drug delivery, yet additional work is needed to prove their safety and efficacy.

## Competing Interests

 The authors declare no known competing financial interests or personal relationships that might have appeared to influence the present work.

## Data Availability

 Data will be made available on request.

## Ethical Approval

 Not applicable.
